# AA-Amyloidosis in the Eurasian stone-curlew (*Burhinus oedicnemus*)

**DOI:** 10.1371/journal.pone.0331573

**Published:** 2025-09-02

**Authors:** Lucía Marrero-Ponce, Cristian M. Suárez-Santana, Óscar Quesada-Canales, Natsumi Kobayashi, Candela Rivero-Herrera, Lucía Caballero-Hernández, Tomoaki Murakami, Antonio Fernández

**Affiliations:** 1 Unit of Veterinary Histology and Pathology, University Institute of Animal Health and Food Safety (IUSA), Veterinary School, University of Las Palmas de Gran Canaria (ULPGC), Spain; 2 Laboratory of Veterinary Toxicology, Tokyo University of Agriculture and Technology, Tokyo, Japan; The University of Texas Health Science Center at Houston, UNITED STATES OF AMERICA

## Abstract

Amyloidosis is a group of protein misfolding diseases and a well-recognized disorder in avian species. However, the knowledge of wild avian amyloid proteome is scarce. We report here gross, histopathological, ultrastructural, immunohistochemical and proteomic findings of systemic amyloidosis in seven Eurasian stone-curlews (*Burhinus oedicnemus*) necropsied in the Canary Islands. Spleen (5/6–83.33%), liver (3/5–60%), kidney (3/5–60%), proventricle (3/5–60%) and intestine (3/6–50%) were the more severely affected organs. All cases underwent chronic inflammatory processes associated to helminth, bacteria or fungi infection. Verminous chronic ventriculitis was the most frequent associated pathology in 5/7 (71.43%) followed by bumblefoot in 2/7 (28.57%) cases. Electron microscopy revealed a predominantly amorphous substance with 10 nm diameter non-branching amyloid fibrils. AA amyloidosis was characterized by immunohistochemistry and mass spectrometry analysis. By mass spectrometry three amyloid signature proteins were also identified: vitronectin, apolipoprotein A-IV and apolipoprotein A-I in 6/7 (85.71%), 4/7 (57.14%), and 3/7 (42.86%) cases, respectively, contributing with new knowledge about the amyloid proteome of amyloidosis in wild avian species.

## Introduction

Amyloidosis is defined as a group of diseases that have in common the pathological deposition of amyloid, an extracellular proteinaceous material derived from the abnormal folding of a whole range of different protein precursors [1,2]. The main constituents of amyloid are insoluble, stable, unbranched fibrillar proteins that self-associate, and are arranged in β-pleated sheets that provide its main property: an orange-to-red color in Congo red stain plus the presence of anomalous color when that stained preparation is seen between two crossed polarizing filters, a polarizer and an analyzer [1–4]. Said fibrils can measure from 7 to 10 nm in diameter and be heterogeneous in length [5]. Frequently, affected organs appear grossly enlarged and firm, with a pale discoloration, while microscopically an extracellular, amorphous, eosinophilic, hyaline substance is observed [2,3,6]. Amyloid deposits can alter the organ structure and function with minimal or absent inflammatory response, sometimes resulting in organ failure [3,7]. Additionally, developing of oligomers occurs in early stages of fibril formation, being proposed their ability to cause cell damage and their possible role as key executors of amyloid diseases [8–11].

Amyloidosis is classified as localized or systemic depending on if the deposits are located near or distant the site of synthesis of the precursor protein, respectively [12]. Amyloid fibril proteins, the primary skeleton of amyloid deposits, are named accordingly to the precursor protein that they arise from, having been characterized 42 different types in human beings [12]. Most amyloid fibrils are rare or very rare, the most frequent types of amyloidosis that can be found in humans are AL (derived from immunoglobulin light chain), wild-type and variant ATTR (derived from transthyretin), and AA (derived from serum amyloid A) [13–15]. In non-human species, 21 different amyloid fibril proteins have been described [16]. The most common type of amyloidosis in domestic, wild and zoo animals, including birds, is AA [7,17,18].

Establishing which type of amyloid fibrils are present in each case is indispensable to know the prognosis of the disease, its proper treatment and even to apply preventive measures [7,19]. Different techniques can be used for typing. Antibody-based approaches, especially immunohistochemistry (IHC), are widely employed, although downsides are common [20,21]. More recently, laser-microdissection (LMD) followed by liquid chromatography-tandem mass spectrometry (LC-MS/MS) has been stablished as the gold standard for amyloid fibril characterization, due to its capability of identifying all proteins present in the deposits, including not only the amyloid fibril implicated but also other proteins associated to it, the amyloid signature proteins (ASPs) [15,22].

In avian species, the type of amyloid deposited has been scarcely determined. Generally, antibody-based methods such as IHC are used [23–27]. In addition, AA amyloid is supposed to lose its affinity for Congo red dye when pretreated with potassium permanganate, as opposed to other amyloid fibrils [28]. This pretreatment has also been employed in cases of avian amyloidosis [29,30]. To pave the way for a deeper understanding of amyloidosis in birds, we studied this disorder in Eurasian stone-curlews (*Burhinus oedicnemus*) from the Canary Islands, applying up-to-date methodologies such as LC-MS/MS. This species is a steppe Paleartic bird that belongs to the order *Charadriiformes*, family *Burhinidae*. Although it is categorized as a Least Concern species by the International Union for Conservation of Nature (IUCN) Red List of Threatened Species, the current population has a declining trend [31]. In the Canarian archipelago two subspecies of Eurasian stone-curlew inhabit, the *B. o. distinctus* in the western islands (Tenerife, Gran Canaria, La Palma, El Hierro and La Gomera) and the *B. o. insularum*, in the more eastern islands (Lanzarote, Fuerteventura, La Graciosa, Lobos and Alegranza) [32]. However, unlike the IUCN status of the species, the Spanish National Catalog of Threatened Species places the *B. o distinctus* in a vulnerable situation and the *B. o. insularum* under special protection rules [33,34]. The Eurasian stone-curlew is considered a suitable “umbrella” species, which are those selected as targets of conservation or protective measures whilst assuming that, indirectly, other not targeted species will also benefit [35]. However, it is of concern the scarce number of studies about the threats that have an impact on the survival of this bird. Many of these works are focused on anthropogenic hazards [36–40], meanwhile, most of the natural diseases reported (of viral [41], bacterial [42], fungal [43] and parasitic etiology [44]) are described in captive specimens not in wild ones. All of this comprise a welfare and conservation problem [10]. Scopus and Web of Science databases have been searched (02/05/2025) with the following keywords: AA amyloidosis, amyloid, amyloid fibrils, amyloid proteome, amyloid signature protein, amyloidosis, avian, avian species, bird, *Burhinus oedicnemus*, immunohistochemistry, proteomics, systemic amyloidosis and wildlife. No reports characterizing the disorder in this species has been found doing these searches, even though the presence of amyloidosis in captive Eurasian stone-curlews has previously been reported [43].

We report seven cases of AA amyloidosis in wild Eurasian stone-curlews of the Canary Islands. Data about macroscopic features, histopathology, ultrastructure, immunohistochemistry and mass-spectrometry proteomics is presented. The presence of AA amyloid fibrils as well as some ASPs is confirmed, and concomitant findings are also described.

## Materials and methods

### Specimen selection, necropsy, tissue sampling and processing

As part of the Wildlife Health Surveillance Network coordinated by the Canarian Government, 74 wild Eurasian stone-curlews (*Burhinus oedicnemus*) were submitted to the University Institute of Animal Health and Food Safety (IUSA) of the University of Las Palmas de Gran Canaria between 2020 and 2022. These specimens were found dead in the wild or died in wildlife recovery centers from the different islands. Body condition of the animals was determined based on pectoral muscle development and subcutaneous and visceral fat deposits [45]. Age of each animal was determined based on gonads development grossly and histologically [46,47].

Specimens with a decomposition status different from fresh and those with no representative or suitable tissue samples for histologic analysis were ruled out of the study, remaining 30 individuals. All of these were necropsied following a standardized and systematic approach [48]. Tissue samples of adrenal glands, central nervous system, esophagus, gizzard, gonads, heart, intestines, kidneys, liver, lungs, pancreas, parathyroid glands, proventricle, skeletal muscle, skin, spleen, thyroid glands and trachea were taken for histologic examination. These were fixed in 4% buffered formalin, routinely processed, embedded in paraffin, cut in 5 µm sections and stained with Hematoxylin-Eosin. A list of the tissue samples taken in each case is provided in S1 Table.

### Microscopic analysis and histochemistry

All tissue samples were evaluated by light microscopy with an Olympus BX51 optic microscope. When the presence of amorphous, eosinophilic, hyaline, extracellular deposits was identified in any tissue, alkaline Congo red stain was performed to all the samples of that case. For the Congo red stain, one tissue section of 6 μm thick per organ evaluated was analyzed in each case. Then, a polarizer (Olympus U-POT) and an analyzer (Olympus U-ANT) were used to verify the existence of an anomalous color birefringence [49]. All slides were evaluated with objectives from 4x to 60x magnification. When a Congo red positive finding with color birefringence was detected, the amyloidosis diagnosis was confirmed. In each organ, amyloidosis was categorized in four groups according to the distribution pattern and severity of the amyloid deposits employing a modification of the method used by Ono et al., (2020) [23]. The categories were (1) mild perivascular deposits, (2) moderate perivascular deposits, (3) severe perivascular deposits with affection of the adjacent parenchyma, and (4) severe perivascular deposits with severe interstitial deposition and disruption of the normal architecture of the organ. The presence of concomitant disorders was also evaluated. The presence of bacteria and fungi in the different tissues was evaluated by direct visualization in the optic microscope plus the use of complementary histochemical stains: Gram, Grocott’s methenamine silver (GMS) and Periodic acid-Schiff (PAS) stains.

### Ultrastructure

For the ultrastructural study, small randomly selected formalin fixed samples of kidney and liver of one animal (case 1) were primarily fixed overnight at 4 ºC in a solution of 2% glutaldehyde in 0.1M phosphate buffer (pH 7.4). Afterwards, the samples were refixed in a solution of 1% osmium tetroxide in 0.1M phosphate buffer (pH 7.4) for 30 minutes. Then, dehydration in graded ethanol series and embedding in Araldite was performed and ultra-thin sections were cut on an LKB ultramicrotome. Semi-thin sections were stained with toluidine blue, while ultra-thin sections were double stained with uranyl acetate and lead citrate. Ultra-thin sections were viewed and photographed under a JEM 1400 transmission electron microscope (TEM; JEOL, Ltd.).

### Immunohistochemistry

Samples of liver, kidney and spleen from all cases except cases 3 and 7, in which no sample of spleen and liver, respectively, were available, were subjected to immunohistochemical analysis. The methodology used was that of Iwaide et al. (2023), in which an in-house anti-SAA antibody, diluted to 1:200, was used as a primary antibody, undiluted horseradish peroxidase-conjugated polymer anti-rabbit IgG antibody (Dako, Santa Clara, California) served as a second antibody, and 3,3’-diaminobenzidine-tetrahydrochloride revealed the positive immunoreactions [50].

### Proteomic analysis

FFPE samples of the liver from cases 1, 3, 5 and 6, and the spleen of cases 2, 4, and 7 were selected for proteomics analysis. The selection was done based on the high abundance of the deposits in these organs. Congo red-positive areas were collected by tissue microdissection and then subjected to LC-MS/MS following previously used protocols [51,52]. Then, the tandem mass spectrometry (MS/MS) results were compared with the theoretical fragmentation patterns of tryptic peptide sequences from a *Charadriiformes* database of proteins registered in the National Center for Biotechnology Information (NCBI) database using the Mascot Server. Significant proteins/peptides were identified using Mascot’s probability-based scoring algorithm. Due to the lack of a specific database of the species addressed in this study (*Burhinus oedicnemus*), protein identities were determined based on the homology with the proteins of other sequenced avian species of their same order (*Charadriiformes*).

## Results

Of the 30 cases included, 7 (23.33%) were diagnosed with amyloidosis. All these specimens were adults, 4 of them were females and 2 were males. The sex in one individual could not be determined. All were admitted in wildlife recovery centers or in veterinary clinics. Detailed information about the signalment of each case is found on Table 1.

**Table 1:** Signalment and gross features of the Eurasian stone-curlews with amyloidosis.

Case	Island	Age	Sex	Body condition	Spleen	Liver	Kidney
1	Gran Canaria	Adult	Male	Thin	X	X	X
2	Lanzarote	Adult	Female	Thin	X	–	–
3	Gran Canaria	Adult	Female	Cachectic	–	X	–
4	Gran Canaria	Adult	Female	Cachectic	X	–	–
5	Tenerife	Adult	Female	Cachectic	X	X	–
6	Gran Canaria	Adult	Male	Thin	X	X	–
7	Lanzarote	Adult	Unknown	Cachectic	X	–	–

Abbreviations: Spleen, splenomegaly; Liver, hepatomegaly; Kidney, nephromegaly; X, presence of splenomegaly, hepatomegaly or nephromegaly; -, absence of splenomegaly, hepatomegaly or nephromegaly.

### Gross and histologic analysis

**Fig 1:**
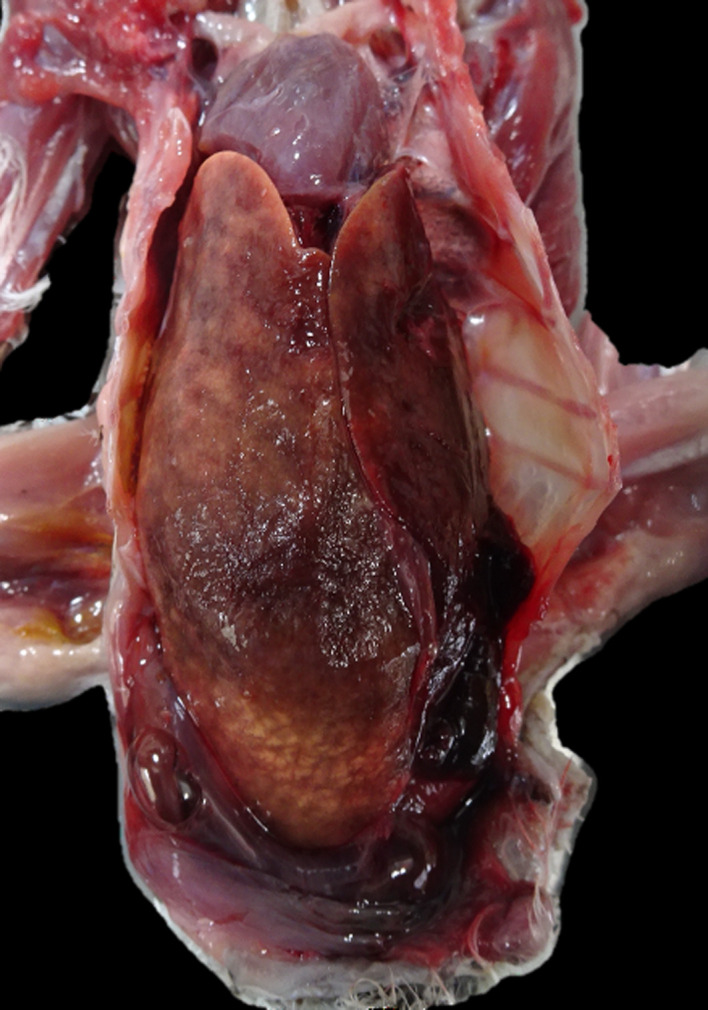
Gross features of amyloidosis, liver, Eurasian stone-curlew (*Burhinus oedicnemus*). Enlarged liver with rounded borders (hepatomegaly) and multifocal to coalescent waxy yellow areas. A blood clot from hepatic laceration is seen in the bottom right area. Case 5.

In the gross evaluation, all specimens were in a poor body condition with muscle atrophy and fat depletion, been classified as cachectic (4/7–57.14% individuals) or thin (3/7–42.86%). A hard to the touch (firm) moderately or severely enlarged liver (hepatomegaly) was observed in 4 animals (cases 1, 3, 5 and 6) with random yellow multifocal to coalescing areas seen in 3 of them (cases 3, 5 and 6) (Fig 1). In case 5, hepatic lacerations in association with a moderate hemocoeloma were observed. Enlargement of the spleen (splenomegaly) ranging from moderate to severe was observed in all cases except case 3. Firm kidneys were present in 2 cases (case 1 and 6), with visible enlargement (nephromegaly) in case 1. Detailed information about the main gross findings of each case is found on Table 1 and a panel comparing healthy spleens, livers and kidneys with enlarged ones is provided in S1 Fig.

**Fig 2:**
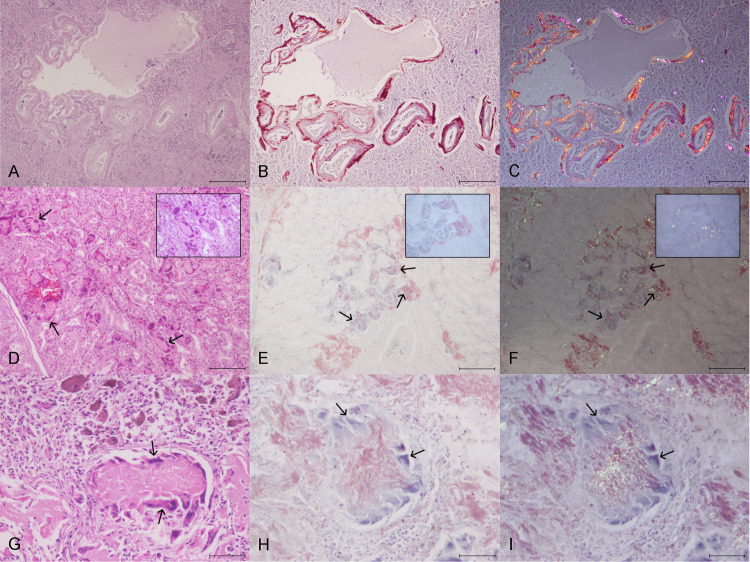
Histopathologic features of amyloidosis in Eurasian stone-curlews (*Burhinus oedicnemus*). **(A-C)** Case 3, kidney, 10x, scale bar is 200 μm. **(A)** Moderate perivascular deposition of amyloid. HE. **(B)** Amyloid deposition observed in Fig 2(A) stained red by Congo red. **(C)** Red and orange birefringence under polarized light. **(D-F)** Case 1, proventricle, 20x, scale bar is 100 μm. **(D)** Multinucleated giant cells engulfing amorphous eosinophilic material (amyloid deposits). Inset: phagocytic cells containing amyloid aggregates, 60x. HE. **(E)** Congo red positive material inside multinucleated giant cells. Inset: phagocytic cells with amyloid aggregates within, 60x. Congo Red. **(F)** Apple green birefringence under polarized light of the Congo red positive material phagocytized. Inset: phagocytic cells with amyloid aggregates within, 60x. (G-I) Case 4, spleen, 40x, scale bar is 50 μm. **(G)** Multinucleated giant cells engulfing amorphous eosinophilic material (amyloid deposits). HE. **(H)** Congo red positive material inside multinucleated giant cells. Congo Red. **(I)** Apple green birefringence under polarized light of the Congo red positive material phagocytized.

Histologically, amyloid accumulation was observed as extracellular, amorphous, eosinophilic, hyaline deposits in 16 of the 18 different organs examined (88.89%) (Fig 2A), all of them showing Congo red positive staining with yellow, green and red birefringence under polarized light (Fig 2B, 2C). Amyloid deposition was more frequently observed in spleen (6/6–100%), skeletal muscle (4/4–100%), intestines (6/7–85.71%), liver (5/6–83.33%), pancreas (4/5–80%), adrenal glands (4/5–80%), heart (5/7–71.43%), kidney (5/7–71.43%), proventricle (5/7–71.43%), ovary (2/3–66.67%) and gizzard (4/7–57.14%). No deposition was found in the trachea and the central nervous system. Regarding to the grade of amyloid deposition among the different affected organs, severe aggregates with marked substitution of the parenchyma were observed in 3/5 (60%) livers, 2/6 (33.33%) spleens, 1/2 (50%) thyroid glands, 1/4 (25%) adrenal gland, 1/4 (25%) pancreas and 1/5 (20%) kidneys. Severe perivascular deposition that extended to the parenchyma with preservation of the organ architecture was seen in 3/5 (60%) proventricles, 3/6 (50%) spleens, 3/6 (50%) intestines, 2/5 (40%) kidneys, 1/4 (25%) gizzards and 1/4 (25%) pancreas. Details of percentage of amyloidosis deposition in each organ evaluated and the grade of deposition observed in each case are illustrated in Table 2. Multinucleated giant cells in the areas of amyloid deposition were seen in the proventricle (case 1), the spleen (case 4) and the liver (cases 1 and 6). However, only in the proventricle (Fig 2D-F) and spleen (Fig 2G-I) the presence of phagocytized amyloid was confirmed.

**Table 2:** Organs evaluated with percentage and grading of amyloid deposition.

Case	In	Ht	Kd	Pv	Gz	Lg	Lv	Sp	Adr	Pcr	Es	CNS	Tr	SM	Th	Ov	Ts	Pth
**1**										n/d					n/d	n/a		n/d
**2**									n/d					n/d			n/a	n/d
**3**								n/d			n/d	n/d	n/d	n/d	n/d		n/a	n/d
**4**															n/d		n/a	n/d
**5**												n/d		n/d		n/d	n/a	
**6**																n/a		n/d
**7**							n/d		n/d	n/d	n/d		n/d			n/d	n/d	n/d
**Overall%**	85.7	71.4	71.4	71.4	57.1	42.9	83.3	100	80	80	40	0	0	100	50	66.7	50	100

Grade of amyloid deposition: Grey, no amyloid deposition; Green, mild perivascular deposits; Yellow, moderate perivascular deposits; Orange, severe perivascular deposit with affection of the adjacent parenchyma; Red, severe perivascular deposits with severe interstitial deposition and disruption of the normal architecture of the organ.

Abbreviations: In, intestines; Ht, heart; Kd, kidney; Pv, proventricle; Gz, gizzard; Lg, lung; Lv, liver; Sp, spleen; Adr, adrenal glands; Pcr, pancreas; Es, esophagus; CNS, central nervous system; Tr, Trachea; SM, skeletal muscle; Th, thyroid glands; Ov, ovaries; Ts, testes; Pth, parathyroid glands; n/d, not determined due to absence of samples; n/a, not applicable referring to reproductive tissues that were not present in the individual.

Concomitant gross and histological findings were present in each case:

Case 1: Detailed findings observed in this specimen have already been published [53]. Briefly, this animal had an amputation of the left pelvic limb with bumblefoot in the contralateral remaining foot, resulting in septicemia, along with vegetative aortic endocarditis and associated myocarditis. A moderate multifocal heterophilic ventriculitis with intralesional nematodes and associated bacterial infection was observed. Numerous cestodes were present in the intestinal lumen without associated inflammation.

Case 2: There were skin and muscle lacerations in the neck, the right shoulder and the right tight, as well as focally extensive necrosis affecting the pectoral muscle with mineralization, histiocytic infiltrate and associated bacterial infection (polyphasic process). Moderate visceral gout was also present affecting pericardium, air sacs, lungs and Glisson’s capsule. There were scarce trematodes in the bile ducts and scarce acanthocephalan in the intestinal lumen.

Case 3: Moderate generalized subcutaneous edema was observed, together with bilateral bone calluses of the furcula. A moderate heterophilic ventriculitis with intralesional nematodes and eggs, associated bacterial infection, as well as marked diffuse thickening and multifocal disruption of the koilin layer were also noted. A focal granuloma with an intralesional cestode was seen in the lungs.

Case 4: The right eye showed a severe ulcerative fibrino-heterophilic keratitis with intralesional fungal hyphae and cocci. A moderate heterophilic ventriculitis with intralesional nematodes and associated bacteria similar to case 3 was observed.

Case 5: A severe heterophilic ventriculitis with intralesional nematodes and associated bacterial infection was diagnosed. A mild lymphoplasmacytic enteritis with cestodes in the intestinal lumen was also seen.

Case 6: A severe heterophilic arthritis of the right intertarsal joint with a focally extensive ulcer on its dorsal area was observed. The foot of the same limb exhibited moderate pododermatitis on the dorsal aspect while mild arthritis was observed in the metatarsal joints of both feet. Scarce cestodes without inflammatory reaction were present in the intestines.

Case 7: Mild histiocytic proventriculitis associated to nematodes with hyperplasia of the mucosal folds and increased mucus production was seen, together with severe heterophilic ventriculitis with intralesional nematodes and associated bacterial infection. Additionally, numerous acanthocephalans were observed in the intestinal lumen.

### Ultrastructure

**Fig 3:**
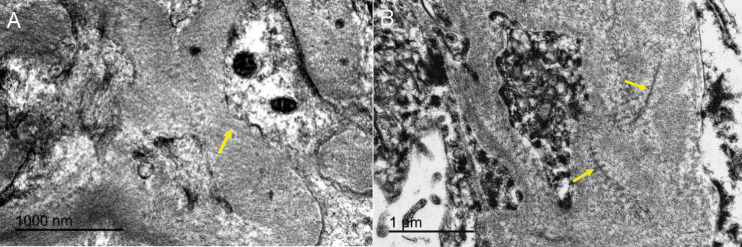
Ultrastructural features of amyloidosis in Eurasian stone-curlews (*Burhinus oedicnemus*). **(A)** Case 1, kidney, scale bar is 1000 nm. Amyloid deposits composed by a large amount of amorphous substance with a scarce amount of randomly arranged non-branching amyloid fibrils (yellow arrow). **(B)** Case 1, kidney, scale bar is 1 μm. Higher magnification of (A) in which scarce amyloid fibrils (yellow arrow) are present.

To confirm the presence of amyloid deposition and for direct visualization of the amyloid fibrils in tissues, an ultrastructure analysis was performed. In the samples visualized by transmission electron microscopy, a predominately amorphous substance with a scarce amount of approximately 10 nm diameter haphazardly arranged non-branching amyloid fibrils was seen displacing normal cells and extracellular material (Fig 3).

### Immunohistochemistry

**Fig 4:**
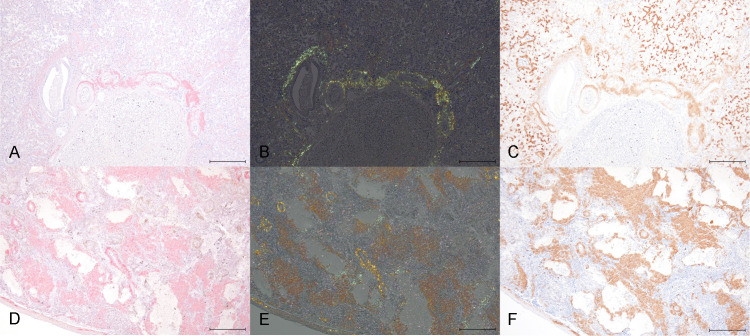
Immunohistochemistry for SAA, Eurasian stone-curlew (*Burhinus oedicnemus*). **(A-C)** Case 1, liver, 10x, scale bar is 200 μm. Moderate perivascular amyloid deposition stained with Congo red **(A)**, seen under polarized light **(B)**, and positively immunolabeled with anti-SAA antibody **(C)**. **(D-F)** Case 6, spleen, 10x, scale bar is 200 μm. Severe perivascular amyloid deposition that extends to the parenchyma stained with Congo red **(D)**, seen under polarized light **(E)**, and positively immunolabeled with anti-SAA antibody **(F)**. Due to three-dimensional variations within the tissue, Fig 4D and 4E do not match exactly with Fig 4F, even though the same area is depicted.

To determine the type of amyloidosis, an immunohistochemical analysis was performed to detect SAA antigens in the affected tissues. All amyloid deposits present in liver, kidney and/or spleen of the seven Eurasian stone-curlew cases showed a strong positive reaction to the anti-SAA antibodies. The immunopositivity, observed as an intense brown staining, was localized at the extracellular level and matched the Congo red positive and birefringent under polarized light material of each sample (Fig 4). No immunolabeling was observed in liver of case 2 and kidney of cases 2 and 7 since no amyloid deposition had been detected previously. Detailed information about anti-SAA antibodies specificity in avian species, as well as about the negative and positive controls of Congo red, polarized light and SAA immunohistochemistry are provided as supporting information in S1 Text.

### Proteomic analysis

In order to obtain the detailed composition of the aggregates, the proteins present in the amyloid deposits were determined by LC-MS/MS. Results from the LC-MS/MS analysis are portrayed in Table 3. The most abundant amyloidogenic protein identified in all cases was serum amyloid A (SAA). Apolipoprotein A-I (ApoA-I) and apolipoprotein A-IV (ApoA-IV), known as amyloidogenic proteins as well as ASPs were present in the deposits of 3/7 (42.86%) and 4/7 (57.14%) cases, respectively. Vitronectin, another ASP was present in all cases but case 1.

**Table 3:** Protein identification by LMD-MS/MS.

Case ID	NCBI Accession	Description	Score	EmPAI
1	PKU49178.1	**Serum amyloid A (*Limosa lapponica baueri*)**	46	0.69
2	KGL98878.1	**Serum amyloid A protein, partial (*Charadrius vociferus*)**	79	0.77
	XP_054064522.1	Vitronectin (*Rissa tridactyla*)	76	0.06
3	KGL98878.1	**Serum amyloid A protein, partial (*Charadrius vociferus*)**	105	1.35
	XP_054078844.1	Apolipoprotein A-IV (*Rissa tridactyla*)	123	0.48
	XP_054064522.1	Vitronectin (*Rissa tridactyla*)	191	0.21
	KGL88706.1	Apolipoprotein A-I, partial (*Charadrius vociferus*)	29	0.11
4	KGL98878.1	**Serum amyloid A protein, partial (*Charadrius vociferus*)**	157	4.20
	XP_054054332.1	**Serum amyloid A protein-like (*Rissa tridactyla*)**	99	1.34
	XP_054064522.1	Vitronectin (*Rissa tridactyla*)	173	0.20
	PKU35349.1	Apolipoprotein A-IV (*Limosa lapponica baueri*)	40	0.08
5	KGL98878.1	**Serum amyloid A protein, partial (*Charadrius vociferus*)**	131	2.00
	KGL88706.1	Apolipoprotein A-I, partial (*Charadrius vociferus*)	287	0.86
	XP_054078844.1	Apolipoprotein A-IV (*Rissa tridactyla*)	154	0.57
	XP_054064522.1	Vitronectin (*Rissa tridactyla*)	198	0.20
6	KGL98878.1	**Serum amyloid A protein, partial (*Charadrius vociferus*)**	141	4.20
	KGL88706.1	Apolipoprotein A-I, partial (*Charadrius vociferus*)	255	0.68
	XP_054054332.1	Serum amyloid A protein-like (*Rissa tridactyla*)	58	0.53
	XP_054078844.1	Apolipoprotein A-IV (*Rissa tridactyla*)	110	0.25
	XP_054064522.1	Vitronectin (*Rissa tridactyla*)	222	0.20
	KGL93419.1	Vitronectin, partial [Charadrius vociferus]	104	0.20
7	PKU49178.1	**Serum amyloid A (*Limosa lapponica baueri*)**	27	0.29
	XP_054064522.1	Vitronectin (*Rissa tridactyla*)	111	0.20

Abbreviations: EmPAI, exponentially modified protein abundance index; NCBI, National Center for Biotechnology Information.

## Discussion

In this article, macroscopic features, histopathology, ultrastructure, immunohistochemistry and proteomics of seven cases of systemic AA amyloidosis in Eurasian stone-curlews are described. AA amyloidosis is produced by diseases that entail long-lasting increased serum amyloid A (SAA) levels, including inflammation, infection, and some neoplasia [54]. Hence, SAA proteins are the precursors of AA amyloid fibrils [14]. SAA are a group of acute phase lipoproteins synthetized mainly by the liver in response to tissue injuries and inflammation, also being demonstrated in humans the role of macrophage-derived cells in their synthesis [55]. Regarding to avian species, domestic and wild, AA amyloidosis is the principal, and almost solely, type of systemic amyloidosis diagnosed. Less commonly detected is the Aβ amyloidosis, found in brains in relation to aging [27] and associated to organochlorine pesticides exposure in raptors [56].

Amyloidosis has been described as an age-related disease in avian species [57]. In concordance with this, all seven specimens with amyloid deposition in this study were adults. In all cases, an inadequate body condition consisting of partially or completed depleted from body fat (visceral and subcutaneous) with moderate to severe pectoral atrophy was observed, which agrees with findings of other studies focused on systemic AA amyloidosis in wild species [58,59]. This poor body condition could be the result of chronic primary diseases, as has been previously described in birds [60], rather than a consequence of amyloidosis itself.

Almost any organ can be affected by amyloid deposition in the case of AA amyloidosis, being spleen, liver and kidney more frequently reported [7,26,59,61]. When amyloid deposits accumulate enough to be perceived macroscopically, enlargement and hardening of the tissue occurs, sometimes with a waxy texture [3]. In these Eurasian stone-curlews splenomegaly was the main macroscopic finding, observed in 6/7 (85.71%) cases. To a lesser extent firm enlarged livers and kidneys were also detected. These gross findings are in concordance with histologic results, being the spleen the most severely affected organ, as stated for other species of the order *Charadriiformes* [59]. Frequent deposits were present in the digestive tract, with extension beyond the perivascular area in many cases. This finding differs with what is reported for humans, where gastrointestinal affection is considered a rare finding in AA amyloidosis [62]. Amyloidosis transmission from fecal amyloid fibrils from cheetahs to mice has been achieved [63], as well as experimental oral transmission of amyloidosis among quails [64]. The presence of amyloid deposition in the digestive tract of cheetahs and quails, respectively, in those studies, added to the finding of frequent amyloid deposition in the digestive tract of Eurasian stone-curlews in this study elicit the possibility of fecal-oral transmission among individuals of this species. Even though the potential horizontal transmission of AA amyloidosis in non-experimental environments has not been confirmed, special careful measures are recommended when managing Eurasian stone-curlews in recovery centers or during conservation programs. The finding of amyloid substance in less frequently reported organs such as esophagus, gonads, thyroid and parathyroid can be attributed to a less thorough sampling in other studies. The presence of amorphous material with a low amount of non-branching fibrils of approximately 10 nm in diameter was stated using transmission electron microscopy in samples of liver and kidney of case 1. This is a feature of amyloid fibrils widely recognized in the literature [3].

In the literature, it has been previously suggested that amyloid deposits do not elicit an inflammatory reaction *per se* [3]. However, macrophages have already been seen near to AA amyloid deposits before [65]. Two roles are proposed for macrophages in relation to amyloidosis: favoring fibril formation [66] and getting rid of deposits [67]. Phagocytosis and clearance of amyloid by these leukocytes have been evidenced in mice [65] while granulomatous infiltrates have been reported in spleens of mute swans [26] and badgers [68] with systemic amyloidosis, with the presence of amyloid in the cytoplasm of macrophages being identified in the last study. In the proventriculus of case 1 and the spleen of case 4 deposits were in association with multinucleated giant cells that had often Congo red positive material within. Hence granulomatous inflammation engulfing amyloid deposits is a rare but possible histological characteristic of AA amyloidosis in the Eurasian stone-curlew. This finding supports the amyloid removal role of macrophages in this species, as could be suggested to happen in other avian species, an important finding in the pathogenesis of the diseases in birds and for the development of potential new therapies.

Avian AA amyloidosis has been mainly described in relation to chronic inflammatory reactions [29,59], especially in waterfowl, usually in association with bumblefoot [26], as well as in the amyloid arthropathy of chickens, generally described as a consequence of *Enterococcus faecalis* infection [69]. It has also been associated to neoplastic diseases [25] and even an idiopathic origin is described [59]. In our study, all individuals presented at least one chronic inflammatory process. The concomitant disorder more often diagnosed in these Eurasian stone-curlews were verminous ventriculitis, with 5/7 (71.43%) animals affected (cases 1, 3, 4, 5 and 7). These individuals showed thickening of the ventricle’s wall, which could be seen macroscopically and/or histologically. Besides, one on the animals also presented a verminous proventriculitis with hyperplasia of the mucosal epithelium. Thickening of the mucosa in cases of nematode infection in the stomach is a well-recognized chronic feature of the infection in birds [70]. Association between helminth infestation and the development of amyloidosis has been previously proposed in different species [71,72], and systemic amyloidosis in relation to chronic ventriculitis caused by nematodes has already been reported in passerine birds [73], which supports the role of the verminous ventriculitis in the appearance of the amyloid deposits in our cases. Besides ventriculitis, case 4 also presented a fungal and bacterial keratitis. SAA levels increase with bacterial stimulation in chicken, quails and falcons [74,75], as well as with fungal infection in falcons [76]. A similar response to infections with these types of agents could also be anticipated in other birds, although some variation can be expected in acute phase responses among species and specific research in Eurasian stone-curlews is lacking. Cases 1 and 6 had pododermatitis in one of their legs because of amputation of the contralateral one and an articular disorder, respectively. Amyloidosis in relation with bumblefoot has not only be reported in Anatidae and other waterfowl but also in other avian species such as raptors [77] and penguins [78], which allow us to suggest that in the genus *Charadriiformes* it could also lead to the development of the disease. In case 2, visceral gout, a consequence of subacute or chronic nephritis in birds [79], was the main chronic process observed. This disease has been significantly associated with AA amyloidosis in raptors [77]. One of the cases (case 2) presented a polyphasic process in the pectoral muscle with mineralization, which is also a sign of chronicity [60]. All seven individuals in this study spent some time in wildlife recovery centers. Although captivity has been proposed as a risk factor for the development of AA amyloidosis in some species such as the island foxes [80], we lack sufficient data to claim the same for the Eurasian stone-curlew.

The type of amyloid fibrils implicated in each case of amyloidosis determine not only the origin of the disease but also its possible treatments and prognosis [81,82]. This fact makes its characterization of utmost importance. Most cases in birds are attributed to AA amyloid deposits, although many are the reports that lack evidence of it [83–87] or that relay in out-of-date methods [25,29,73]. Antibody-based techniques, mainly IHC, have been used as the main method to characterize amyloid fibrils in avian species [23,24,26]. In our cases, samples of liver, kidney and/or spleen of each case, when they showed Congo red positive staining of amyloid deposits, also displayed a strong positive reaction using the anti-SAA antibody, confirming the presence of AA amyloidosis in the specimens.

Despite IHC capacity to determine whether a suspected antigen is present in the sample or not, many drawbacks exist, such as the availability of antibodies for the least common forms of amyloidosis, the sensibility and specificity of antibodies, the possibility of non-specific staining and the need of a different test for each type of suspected amyloid fibril [20,81]. These downsides accentuate in cases of scanty studied wild species, such as the Eurasian stone-curlew, in which many unique information can be lost. Since approximately two decades, the concept of One Health has become increasingly important. This term is based in the interconnection of human, animal and environmental health, promoting interdisciplinary collaborations and deepening in knowledge that can be of use to different sectors [88]. Due to this, the concern about wildlife’s diseases has also grown significantly, not only in respect to zoonotic diseases but in matters of conservation approaches. All of this raise the importance of addressing the unknown with cutting-edge unequivocal techniques. This is why we decided to also apply mass-spectrometry analysis, which allows the identification of all proteins present in the amyloid deposits and has been established as the gold standard for amyloidosis typification [19].

Typification by LC-MS/MS was applied to all cases, where the liver or the spleen were analyzed based on the abundance of amyloid deposits to be dissected. When various amyloid-forming proteins are present in the deposits, the one most quantified determines the type of amyloidosis [13]. SAA was present in all samples, always showing a higher EmPAI than the other amyloid forming proteins. Therefore, AA amyloidosis was diagnosed in all cases. ApoA-I and ApoA-IV were also detected in 3 and 4 specimens, respectively. However, it is important to bear in mind that these apolipoproteins, among others, not only are able to produce amyloidosis diseases but can also be part of the microenvironment of amyloid deposits acting as ASPs [13]. To render a diagnose of ApoA-I (AApoAI) or ApoA-IV amyloidosis (AApoAIV), this precursor proteins should be abundant in the deposits meanwhile other amyloid precursor proteins should present a low or non-existent quantity [16]. This does not occur in our Eurasian stone-curlew, hence ApoA-I and ApoA-IV would act as ASPs in these cases. With the use of proteomic characterization of amyloid deposits, ASPs are increasingly used to confirm that the samples evaluated do contain amyloid deposits [89]. The intricated combination of peptides and proteins that constitute amyloid deposits in humans has been widely studied, allowing the use of LC-MS/MS for their categorization as: fibril forming proteins; fibril forming as well as non-fibrillar constituents; non-fibril forming proteins; and contaminants [13]. The most reported ASPs in humans are ApoA-IV, ApoA-I, apolipoprotein E (ApoE), serum amyloid P component (SAP), heparan sulphate proteoglycans (HSGPs) and vitronectin (VTN) [13,90]. Information about ASPs in the veterinary literature is scare, although some information in felines, dogs and foxes exists [51,52,91,92]. Based on the databases search previously mentioned, no reports of avian ASPs were found. We detected the presence of vitronectin, ApoA-I and ApoA-IV in these Eurasian stone-curlews. No SAP, ApoE or HSGPs were present in any of the deposits, even though SAP has been reported as present in all forms of amyloidosis [6]. ASPs found in our specimens compared with those in other animal species already studied indicate that the amyloid microenvironment varies among species, and even some variation is observed between individuals of the same species. These variations could be an indicator of differences in the pathogenesis of the disease among species or genetics among individuals.

It must be mentioned that no information about the peptide sequences of the Eurasian Stone-curlew proteins is available, so no databases to match the LC-MS/MS data with exist. For that reason, protein identification in this study was done based on peptide homology with other species of the order *Charadriiformes* present in a pre-existent database, which could result in lower score and EmPAI results in the proteins identified.

This work characterizes the disorder of AA amyloidosis in Eurasian stone-curlews and provide new valuable information in the field of avian amyloidosis in general. We conclude that the amyloid deposits in the Eurasian stone-curlew with systemic AA amyloidosis are composed by SAA, vitronectin, ApoA-I and ApoA-IV. Further research is encouraged to determine whether these findings are consistent across other avian species.

## Supporting information

S1 Table Tissue samples collected for histological analysis from each Eurasian stone-curlew evaluated. The “X” is used to mark the tissue sample fixed in formalin. Blank spaces indicate no samples of those tissues were collected.
(XLSX)

S1 Fig Size reference of healthy & enlarged organs. Healthy spleen (A), liver (C) and kidneys (E), and enlarged spleen (B), liver (D) and kidneys (F). Scale bar is 1 cm in each image.
(TIF)

S1 Text Antibody specificity, negative and positive controls. Brief explanation of the anti-SAA antibody specificity in birds, as well as the negative and positive controls used in the histochemical analysis.
(PDF)
