# Supplementary figures and images for: AA-Amyloidosis in the Eurasian stone-curlew (Burhinus oedicnemus)
Source: PLoS One. 2025 Sep 2;20(9):e0331573. doi: 10.1371/journal.pone.0331573 (PMC12404442; doi:10.1371/journal.pone.0331573)

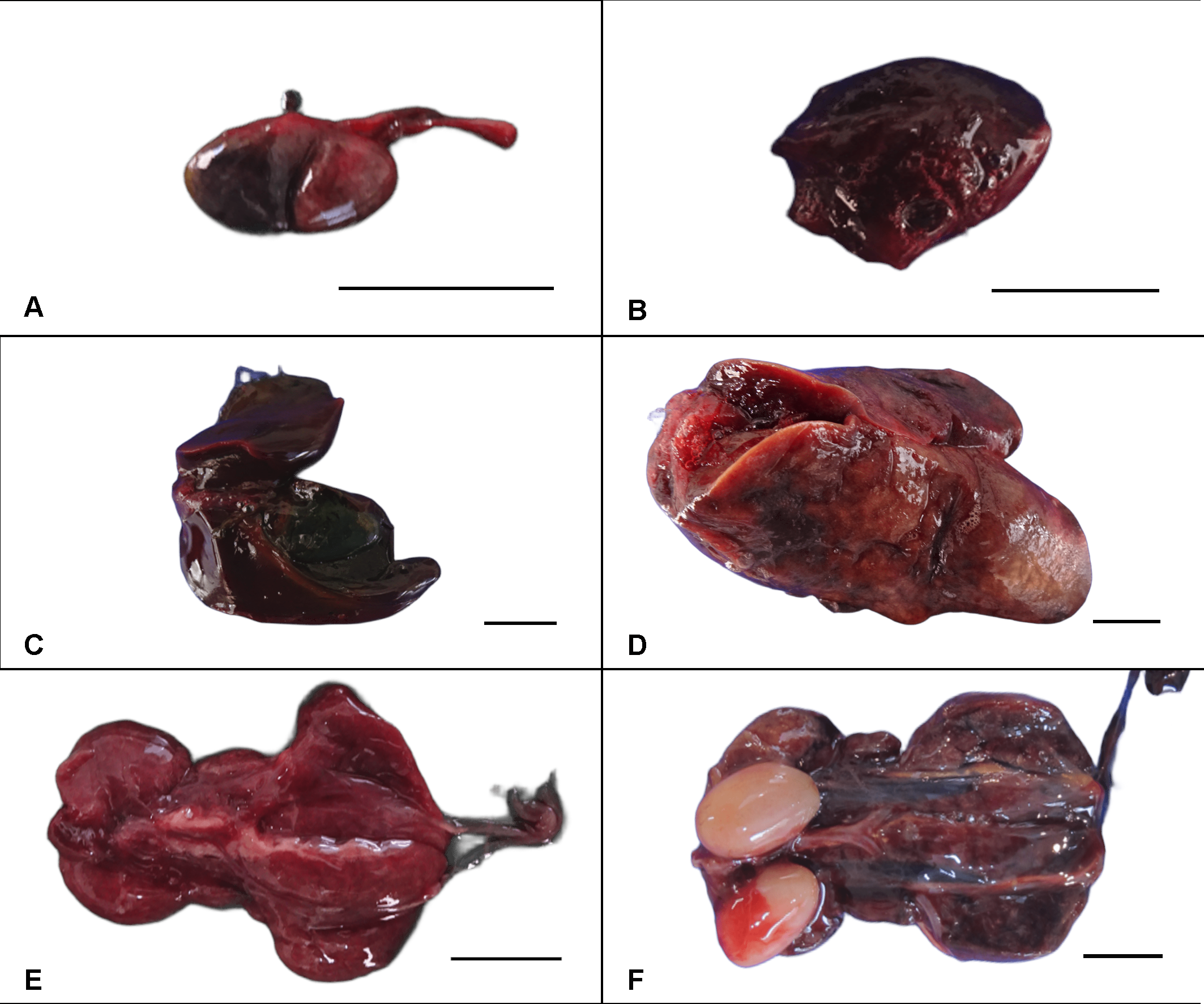

Supplement: S1 Fig — Healthy spleen (A), liver (C) and kidneys (E), and enlarged spleen (B), liver (D) and kidneys (F). Scale bar is 1 cm in each image. (TIF) [file pone.0331573.s002.tif]
