# Supplementary material for: AA-Amyloidosis in the Eurasian stone-curlew (Burhinus oedicnemus)
Source: PLoS One. 2025 Sep 2;20(9):e0331573. doi: 10.1371/journal.pone.0331573 (PMC12404442; doi:10.1371/journal.pone.0331573)
Supplement: S1 Text — Brief explanation of the anti-SAA antibody specificity in birds, as well as the negative and positive controls used in the histochemical analysis. (PDF) [file pone.0331573.s003.pdf]

The in-house antibody used in this article was produced using an antiserum obtained immunizing against SAA at Asp32-Gly50 residue, which is highly conserved across species. This has been verified by using a mass spectrometry analysis and a table depicting de SAA sequence in cats, great cormorant and birds of order Charadriiformes (same order as the *Burhinus oedicnemus*) is provided (Table 1). The utility of the in-house anti SAA antibody has been verified, and a paper using it has been published (1).

Species	Accession (NCBI or Uniprot)	SAA sequence (Asp32-Gly50)
Cat	P19707	DKYFHARGNYDAAQRGPGG
Great cormorant	XP_009503745.2	DKYFHARGNYDAAQRGPGG
Limosa lapponica baueri	PKU49178.1	DKYFHARGNYDAAQRGPGG
Killdeer	KGL98878.1	DKYFHARGNYDAAQRGPGG
Black-legged kittiwake	XP_054054332.1	DKYFHARGNYDAAQRGPGG

Table 1. Alignment of the SAA sequence, which was used for the in-house SAA antibody production.

For our article, cat tissue was used as negative and positive controls of Congo red, polarized light and SAA immunohistochemistry (Figure 1). In order to provide more evidence of the in-house antibody specificity in avian species, immunopositivity of tissue from a Great cormorant was also verified (Figure 2).

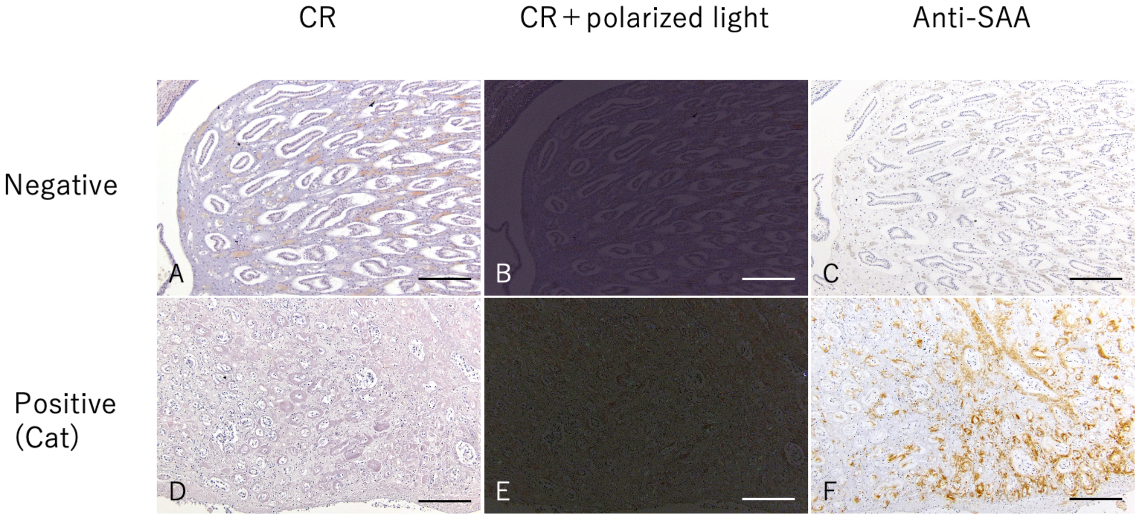

Figure 1. Congo red, polarized light and Anti-SAA negative and positive controls. (A-C) Cat kidney with no amyloid deposition. (D-F) Cat kidney with amyloid deposition. Scale bar is 200 μm

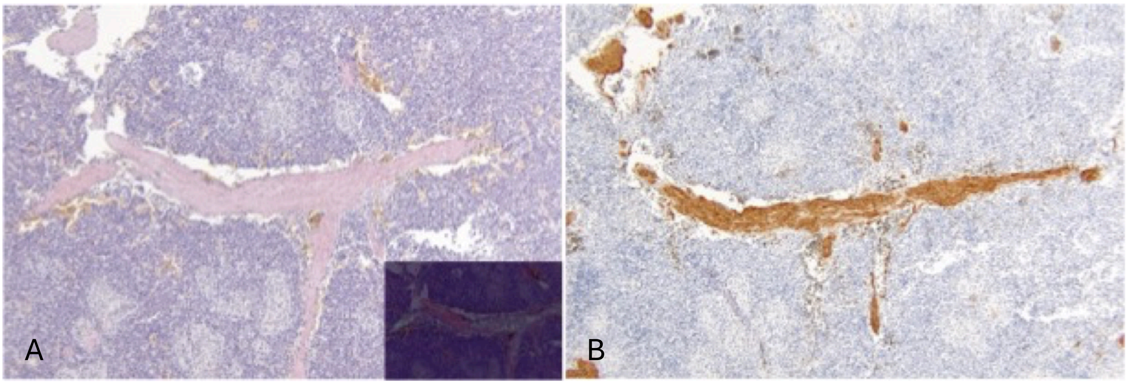

Figure 2. Congo red and immunohistochemistry in Great cormorant, Spleen. (A) Congo red staining in the spleen of a Great cormorant with amyloid deposition. Inset: birefringence under polarized light. (B) Amyloid aggregates in the trabeculae and veins shows strong positive for anti-SAA antibody.

References:

１. Kobayashi N, Kaneda M, Ikeda M, Kondo H, Iwaide S, Itoh Y, et al. Polymorphisms in SAA alter intrarenal amyloid distribution of AA amyloidosis in cats. Scientific reports. 2025; 15: 21553. doi: 10.1038/s41598-025-07983-7
